# Editorial: Women in systems neuroscience

**DOI:** 10.3389/fnsys.2025.1576398

**Published:** 2025-04-14

**Authors:** Jacqueline K. Rose, Susan Shore, Lili Li

**Affiliations:** ^1^Behavioral Neuroscience Program, Department of Psychology, Western Washington University, Bellingham, WA, United States; ^2^Otolaryngology, Molecular and Integrative Physiology, University of Michigan, Ann Arbor, MI, United States; ^3^College of Health Science and Environmental Engineering, Shenzhen Technology University, Shenzhen, Guangdong, China

**Keywords:** auditory, behavior, modeling, systems neuroscience, computational

## 1 Introduction

In the field of scientific research, gender disparities bring many challenges for women scientists, including implicit biases in research and publishing. Marie Curie made groundbreaking contributions not only to research but also to the progress of women in scientific fields. As the first woman to win a Nobel Prize, she broke barriers in a male-dominated field. Her success demonstrated that women could also achieve the highest levels of scientific endeavor.

Over the past decades, System Neuroscience has become a critical subfield for exploring the mechanisms underlying behavior and cognition. Many women neuroscientists have made outstanding contributions to this field. Dr. Eve Marder revealed the principles of neuromodulation and the dynamics of small neural networks (see Nassim, 2018). Dr. Sandhya Koushika developed novel techniques for studying axonal transport (Murthy et al., 2011). Dr. Mary Jeanne Kreek pioneered innovative treatments for drug addiction (Stimmel and Kreek, 2000). Moreover, Dr. Yasmin Hurd disclosed the epigenetic and cellular mechanisms underlying addiction (Yevoo and Maffei, 2022). However, contributions of women neuroscientists are still underrepresented. This Research Topic, *Women in Systems Neuroscience*, provides a platform to highlight women neuroscientists' significant contributions to Systems Neuroscience.

## 2 Auditory systems

In this volume, in the peripheral auditory system, Lozier et al. identified that there is more fast-gating AMPAR subunits mRNA in females than in males. This could explain the more temporal precise synaptic transmission and increased spiral ganglion synchrony in female mice compared to males. In the central auditory system, Rogalla et al. developed an optogenetic implant in the inferior colliculus to demonstrate the principle of subcortical differential stimulation of sensory systems using complex artificial cues in freely moving animals. They showed that neurons in this subcortical structure were able to respond to optogenetic stimulation as well as auditory stimulation and were able to distinguish between the two types of stimulation.

## 3 Behavior systems

Arce-McShane et al. contributed to the understanding of cortical control of mastication, specifically bite force and static gape in female rhesus macaques. By recording from implanted microelectrode arrays, Arce-McShane et al. reported that bite force showed stronger individual neuron activation in three sub-regions of the orofacial sensorimotor cortex (primary motor, primary somatosensory, and cortical masticatory areas), whereas neuron population activity revealed more signaling variance was accounted for by gape. This study further characterizes how these three areas of the orofacial sensorimotor cortex signal individually and via coordinated population activity in producing oral behaviors and further elucidates the complex cortical signaling patterns that control mastication.

Furriel et al. outlined an original computational model that incorporates signaling from identified fear and stress brain structures (e.g., amygdala). Variations from hormones, as well as both spiking neural networks and integrate-and-fire neurons, were included to predict physiological and behavioral outcomes of fear and stress resulting from stress-enhanced fear learning (SEFL) and immediate-extinction deficit (IED) *in vivo* training. Initially validated using the contextual fear conditioning paradigm, this computational framework predicted the persistence of fear memory as a function of the timing of extinction training following conditioning. The resultant model provides a plausible *in silico* approach to behavioral fear testing to reveal conditions and possible interventions for fear-based disorders.

## 4 Computational systems

A method paper by Jarne described a recurrent neural network (RNN) model to predict neural computation for a simple working memory task (a 3-bit flip-flop). Jarne detailed differential equations, discretization, task parameterization, and analysis methods for the resultant network activity from different inputs. This paper provides additional support for the use of RNN to predict and understand complex neural processes.

## 5 Conclusion

Female researchers in the field of systems neuroscience have made significant advances in key areas. Their studies have not only revealed the complex functions of the brain but also provided substantial support and inspiration for promoting gender equality in scientific research.
